# Multi-biomarker score model for predicting fatal outcomes in severe fever with thrombocytopenia syndrome: a multicenter cohort study

**DOI:** 10.3389/fcimb.2025.1681470

**Published:** 2025-11-17

**Authors:** Mengsha Chen, Yingying Yuan, Tao Ju, Rongrong Qu, Siheng Zhu, Airong Hu, Zumo Zhou, Lina Xu, Piao Hu, Yejin Xu, Lianqing Lou, Shibo Li, Wei Ye, Shigui Yang, Dong Yan

**Affiliations:** 1 Department of Emergency Medicine, Second Affiliated Hospital; Department of Epidemiology and Biostatistics, School of Public Health; The Key Laboratory of Intelligent Preventive Medicine of Zhejiang Province, Zhejiang University School of Medicine, Hangzhou, China; 2 State Key Laboratory for Diagnosis and Treatment of Infectious Diseases; National Clinical Research Center for Infectious Diseases; National Medical Center for Infectious Diseases; Collaborative Innovation Center for Diagnosis and Treatment of Infectious Diseases; The First Affiliated Hospital, Zhejiang University School of Medicine, Hangzhou, China; 3 The Quzhou Affiliated Hospital of Wenzhou Medical University, Quzhou People’s Hospital, Quzhou, China; 4 Ningbo No. 2 Hospital, Ningbo, China; 5 Department of Infectious Diseases, Zhuji People’s Hospital, Shaoxing, China; 6 The First People’s Hospital of Xiaoshan District, Xiaoshan Affiliated Hospital of Wenzhou Medical University, Hangzhou, Zhejiang, China; 7 Jinhua Central Hospital, Jinhua, Zhejiang, China; 8 Department of Critical Care Medicine, Yiwu Central Hospital, Jinhua, Zhejiang, China; 9 Wenzhou Medical University Affiliated Zhoushan Hospital, Zhoushan, China; 10 Department of Infectious Disease and Liver Disease, The Second Hospital of Nanjing, Affiliated to Nanjing University of Chinese Medicine, Nanjing, China

**Keywords:** severe fever with thrombocytopenia syndrome, SFTS, multi-biomarker score, prognostic model, mortality, fatal outcome

## Abstract

**Background:**

Severe fever with thrombocytopenia syndrome (SFTS) is a rapidly progressive disease with high mortality. This study aims to identify mortality risk factors in SFTS and create a prognostic model for early high-risk patient identification.

**Methods:**

A total of 301 SFTS patients were enrolled between May 2012 and July 2023 from nine clinical centers across the East China region. Using principal component analysis and cox regression, we identified independent risk factors for mortality and constructed the multi-biomarker score model (1 point was assigned when age ≥60 years, AST/ALT ratio ≥2.23, BUN ≥5.6 mmol/L, ALP ≥ 68 U/L, or ALB <33.3 g/L).

**Results:**

Of the 301 patients with SFTS, 57 (18.9%) experienced fatal outcomes during hospitalization. The risk of mortality escalated with each additional point on the multi-biomarker score, with a HR of 2.04 (95% CI, 1.60–2.61). Patients were stratified into low (0–1), intermediate (2–3), and high (4–5) risk groups based on their multi-biomarker scores. Notably, those in the high-risk category were at an over eightfold increased risk of mortality (HR, 8.59; 95% CI, 2.63–28.05). High scores (4–5) were also predictive of adverse outcomes, including secondary bacterial infection, meningitis, ICU admission, heart failure, respiratory failure, and renal failure. The receiver operating characteristic (ROC) curve analysis for the multi-biomarker score revealed an AUC of 0.769 (95% CI, 0.632–0.787), suggesting a cutoff value above 3 as the threshold for optimal discrimination.

**Conclusions:**

SFTS patients with more than three of the following criteria—age ≥60 years, AST/ALT ratio ≥2.23, BUN ≥5.6 mmol/L, ALP ≥68 U/L, or ALB <33.3 g/L—show a significantly elevated mortality risk.

## Introduction

Severe fever with thrombocytopenia syndrome (SFTS) is an emergent tick-borne infectious disease caused by the Dabie bandavirus or SFTS virus (SFTSV), classified within the family Phenuiviridae of the order Bunyavirales (Xue-Jie et al., 2011). SFTSV, first identified in China in 2009, has spread extensively throughout China and has also been reported in several East and Southeast Asian nations, including Japan (Takahashi et al., 2014), South Korea (Kim et al., 2013), Vietnam (Tran et al., 2019), and Myanmar (Aye Marlar et al., 2020), as well as in the United States (McMullan et al., 2012).

Clinically, SFTS is characterized by fever, leukopenia, and thrombocytopenia, alongside gastrointestinal symptoms, myalgia, and headache. Notably, elderly individuals and those with pre-existing health conditions tend to exhibit more severe disease presentations (Liu et al., 2014). In critical cases, patients may succumb to multiple organ dysfunction and death. Research indicates that the mortality rate associated with SFTS ranges from 12% to 50% (He et al., 2024). Given its widespread geographic presence and significant mortality rates, SFTSV poses a substantial threat to global public health.

However, at the time of admission, the clinical manifestations of SFTS are often non-specific, which poses challenges for the prognosis of patients. Specifically, while some patients may exhibit similar symptoms, their prognoses can vary significantly; some may be self-resolving, whereas others can rapidly develop to severe conditions, potentially leading to death (Li et al., 2021). This discrepancy partly arises from individual variations in immune responses and underlying health conditions. Currently, the management of SFTS remains largely supportive, with limited antiviral options available for SFTSV. Studies have indicated that the commonly used treatment ribavirin has little effect on reducing fatal clinical outcomes associated with SFTS (Liu et al., 2013). Therefore, early identification of mortality risk in SFTS patients is crucial, as timely intervention could potentially improve outcomes for those deemed high risk.

However, analyses of high-risk factors for mortality in SFTS are still in the exploratory phase. There exists a scarcity of validated tools for individual-level risk stratification. Recent developments by researchers, such as Qionghan He et al., have proposed several predictive models for mortality risk in SFTS patients (Li et al., 2017; Wei et al., 2022; Fang et al., 2024; He et al., 2024). Nonetheless, these models face significant limitations, including small sample sizes, reliance on single-center data, and complex methodologies that hinder practical application.

Therefore, this study aims to investigate the high-risk factors associated with mortality in SFTS and to develop a straightforward and feasible risk model for identifying high-risk patients at the time of admission, to support for secondary prevention in patients with SFTS.

## Methods

### Data collection

Participants in this study were recruited from nine clinical centers across the East China region between May 2012 and July 2023, including the First Affiliated Hospital of Zhejiang University, The Quzhou Affiliated Hospital of Wenzhou Medical University, Ningbo No. 2 Hospital, Zhuji People’s Hospital, Xiaoshan Affiliated Hospital of Wenzhou Medical University, Jinhua Central Hospital, Yiwu Central Hospital, Wenzhou Medical University Affiliated Zhoushan Hospital, and the Second Hospital of Nanjing Affiliated to Nanjing University of Chinese Medicine. A total of 329 patients diagnosed with SFTS were initially identified. After excluding those without baseline laboratory data or outcome information, 301 patients with complete records were included in the final analysis (**Supplementary Figure 1**). Relevant medical records were obtained from the electronic medical record system. This study was conducted according to the Helsinki II Declaration and approved by the Clinical Research Ethics Committee of the First Affiliated Hospital, Zhejiang University School of Medicine ([2025B] IIT Ethics Approval No.0298).

### Assessment of variables

In this study, we collected the following variables: demographic characteristics, disease history, clinical characteristics, routine laboratory examinations, and treatment regimens. Specifically, these variables included sex, age, history of exposure, medical history of hypertension and diabetes, treatment with glucocorticoids and/or antiviral therapy, and a comprehensive panel of routine laboratory results. The laboratory parameters encompassed hematological, biochemical, and coagulation indicators, including red blood cell (RBC) count, white blood cell (WBC) count, platelet (PLT) count, lymphocytes, monocytes, neutrophils, and atypical lymphocytes; alanine aminotransferase (ALT), aspartate aminotransferase (AST), alkaline phosphatase (ALP), amylase, lipase (LPS), total bilirubin (TBil), total protein (TP), albumin (Alb), and globulin (Gb); renal and inflammatory markers including creatinine (Cr), blood urea nitrogen (BUN), C-reactive protein (CRP), and procalcitonin (PCT); coagulation parameters such as international normalized ratio (INR), activated partial thromboplastin time (aPTT), prothrombin time (PT), thrombin time (TT), D-dimer, and fibrinogen (FIB); as well as additional indicators including urinary protein (proteinuria), hematuria, fecal occult blood, lactate dehydrogenase (LDH), hydroxybutyrate dehydrogenase (HBDH), creatine kinase-MB (CK-MB), cardiac troponin (cTn), and brain natriuretic peptide (BNP). Among these, only age and routine laboratory results were treated as continuous variables; all other variables were classified as categorical. The routine laboratory tests were performed within the first 24 hours following admission to the hospital.

The diagnostic criteria for SFTS were as follows: 1) an acute fever (temperature >37.5 °C for more than 24 hours) accompanied by thrombocytopenia (platelet count <100×10^9/L); and 2) laboratory confirmation of SFTSV infection through detection of viral RNA using fluorescent probe PCR (BGI-GBI, Hangzhou, China), and/or the presence of virus-specific IgM antibodies in peripheral blood, assessed via ELISA (Zhongshan Bio-Tech Co., Ltd., Guangdong, China) (Lin et al., 2019). The primary outcome was survival status, categorized into two groups: improvement leading to discharge and in-hospital mortality, with patient discharge marking the termination of follow-up. The secondary outcome included adverse events of secondary bacterial infection, meningitis, ICU admission, heart failure, respiratory failure, and renal failure.

### Statistical analyses

Categorical variables were reported as frequencies and percentages, and analyzed using the chi-square test. Continuous variables were assessed for normal distribution by Q-Q plots. Normally distributed variables were presented as mean (standard deviation) and compared using t-test; whereas non-normally distributed variables were expressed as median (interquartile range) and compared using the Mann-Whitney test. For routine laboratory measurements with missing values, data imputation was performed using the random forest methodology.

Principal component analysis (PCA) was employed to analyze the distribution patterns of risk factors among SFTS patients, categorized by survival outcomes. This analysis facilitated the identification of key variables contributing to the observed patterns, ranked according to PCA loadings. The five biomarkers with the highest PCA loadings were selected and categorized into binary variables based on their median levels: age (≥60 years), AST/ALT (≥2.23), BUN (≥5.6 mmol/L), ALP (≥68 U/L), and Alb (≥33.3 g/L). Furthermore, Kaplan-Meier survival analysis and Cox proportional hazards models were utilized to compare mortality rates and hazard ratios among patients with varying biomarker levels (low versus high).

The multi-biomarker score assigns 1 point to each biomarker that meets the following criteria: age (≥60 years), AST/ALT (≥2.23), BUN (≥5.6 mmol/L), ALP (≥68 U/L), and Alb (<33.3 g/L), while biomarkers that do not meet these standards receive 0 points. The total score was computed, yielding a multi-biomarker score ranging from 0 to 5, which was subsequently categorized into low (0-1), intermediate (2-3), or high (4-5) risk groups. The association between the multi-biomarker score and both 1) all-cause mortality and 2) adverse outcomes (including secondary bacterial infection, meningitis, ICU admission, heart failure, respiratory failure, and renal failure) was examined using Kaplan-Meier survival analysis and multivariable Cox proportional hazards models. Both unadjusted and adjusted Cox models were employed, with adjustments made for age and sex. In sensitivity analysis, the multi-biomarker score was treated as a continuous variable to explore its association with all-cause mortality across various subgroups. To further assess the robustness of the score components, we employed restricted cubic spline regression to identify optimal cut-off values for each biomarker, replacing the use of median thresholds. Additionally, a weighted biomarker score was constructed based on loading contributions derived from PCA.

All statistical analyses were conducted using R statistical software version 4.3.2 (R Foundation). A two-sided significance level of P < 0.05 was considered statistically significant for all tests.

## Results

### Baseline characteristics of participants

In this study, we identified 301 participants diagnosed with SFTS. Among them, 244 patients (81.1%) were discharged in stable condition, while 57 patients (18.9%) succumbed to the illness during their hospitalization. No significant differences were observed between the survival and non-survival groups in terms of sex, exposure history, disease history, or treatment regimen (**Supplementary Table 1**). Notably, the non-survivor group had a higher mean age compared to the survivor group.

### Independent risk factor for non-survival status among SFTS patients

**Figure 1:**
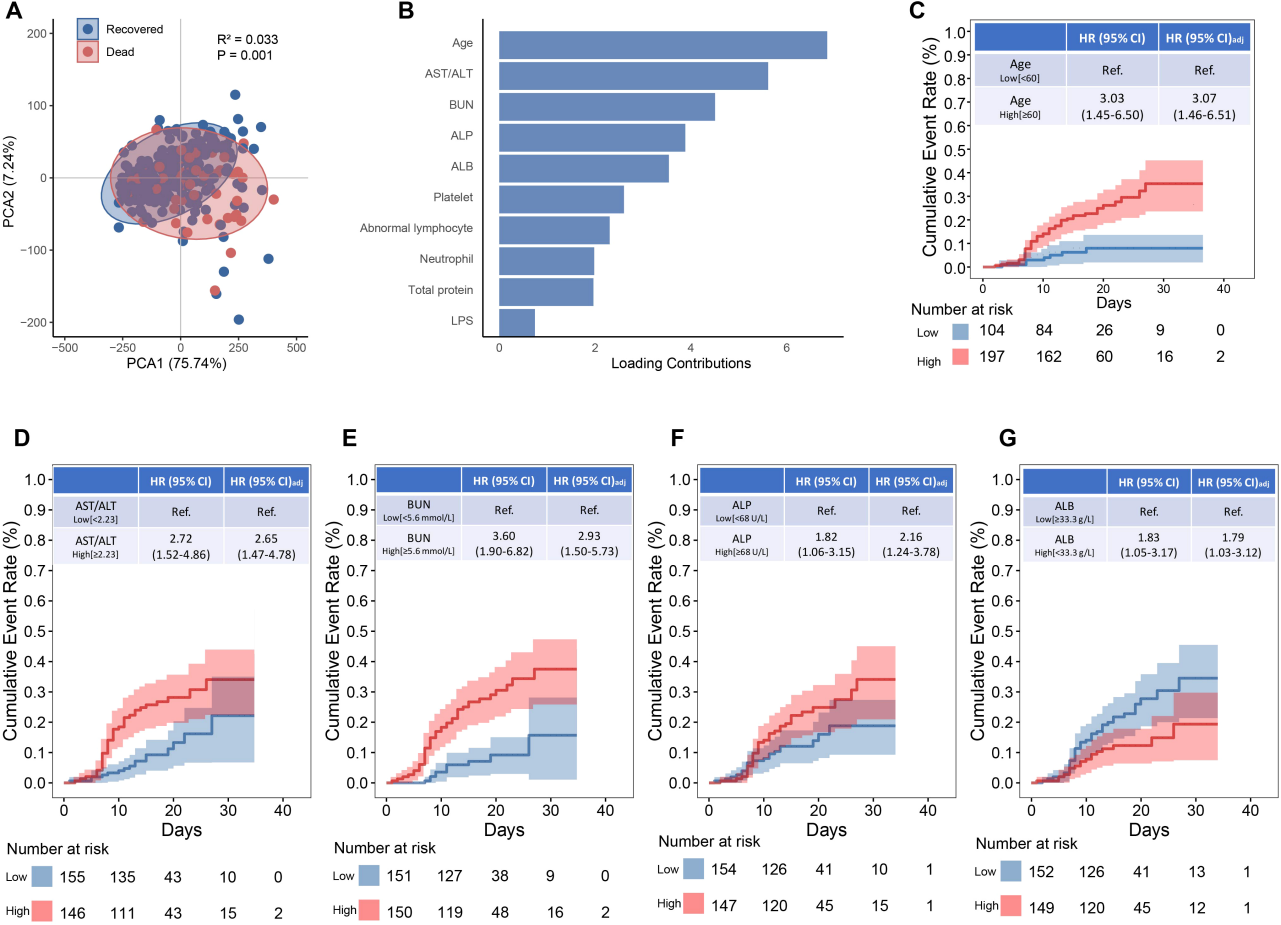
Biomarkers associated with mortality among SFTS patients. **(A)** Principal component analysis between recovered and non-surviving SFTS patients. **(B)** Top 5 contributing factors to the distinct profile. **(C–G)** The Kaplan-Meier estimates of cumulative rates of mortality predicted by age, AST/ALT, BUN, ALP and ALB.

Principal component analysis revealed a distinct separation of laboratory risk factors between the non-survival and survival groups (P = 0.001; **Figure 1A**). Among these, age, AST/ALT, BUN, ALP, ALB, platelet count, abnormal lymphocyte percentage, neutrophil count, total protein, and LPS were identified as the top 10 drivers of the non-survival pattern (**Figure 1B**). Subsequent Kaplan-Meier survival analysis and Cox proportional hazards modeling identified five markers as independent predictors of fatal outcomes in SFTS patients (**Figures 1C–G**): age 60 or above (adjusted HR: 3.07; 95% CI, 1.46-6.51), AST/ALT ratio 2.23 or higher (aHR: 2.65; 95% CI, 1.47-4.78), BUN 5.6 mmol/L or higher (aHR: 2.93; 95% CI, 1.50-5.73), ALP 68 U/L or higher (aHR: 2.16; 95% CI, 1.24-3.78), and albumin below 33.3 g/L (aHR: 1.79; 95% CI, 1.03-3.17).

### Construction of multi-biomarker score for non-survival status

**Figure 2:**
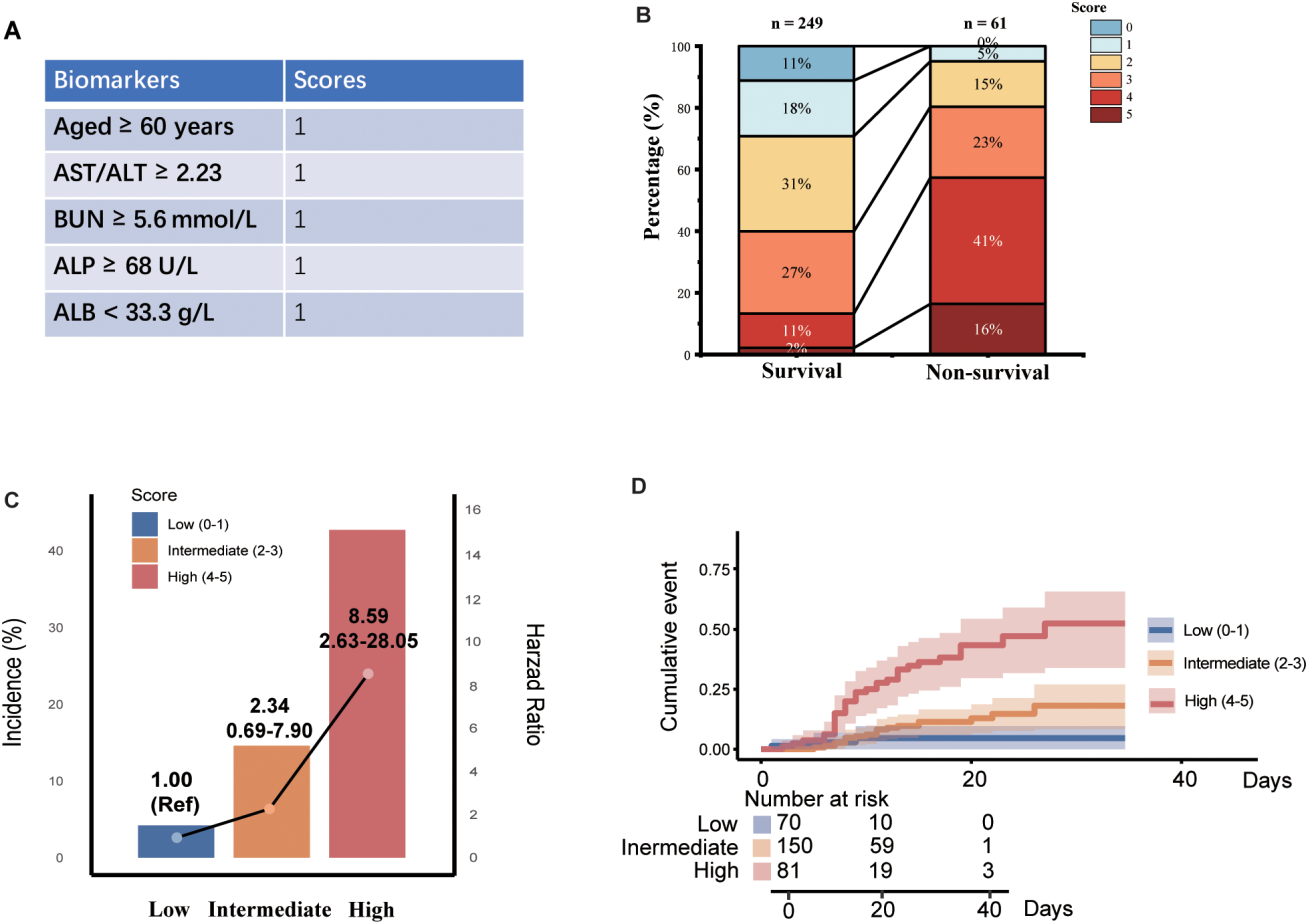
Associations between multi-biomarker score with mortality among SFTS patients. **(A)** Development of multi-biomarker score. **(B)** Distribution of multi-biomarkers among cured and non-surviving group. **(C)** Associations between multi-biomarker score and mortality. **(D)** The Kaplan-Meier estimates of cumulative rates of mortality predicted by multi-biomarker score.

**Figure 3:**
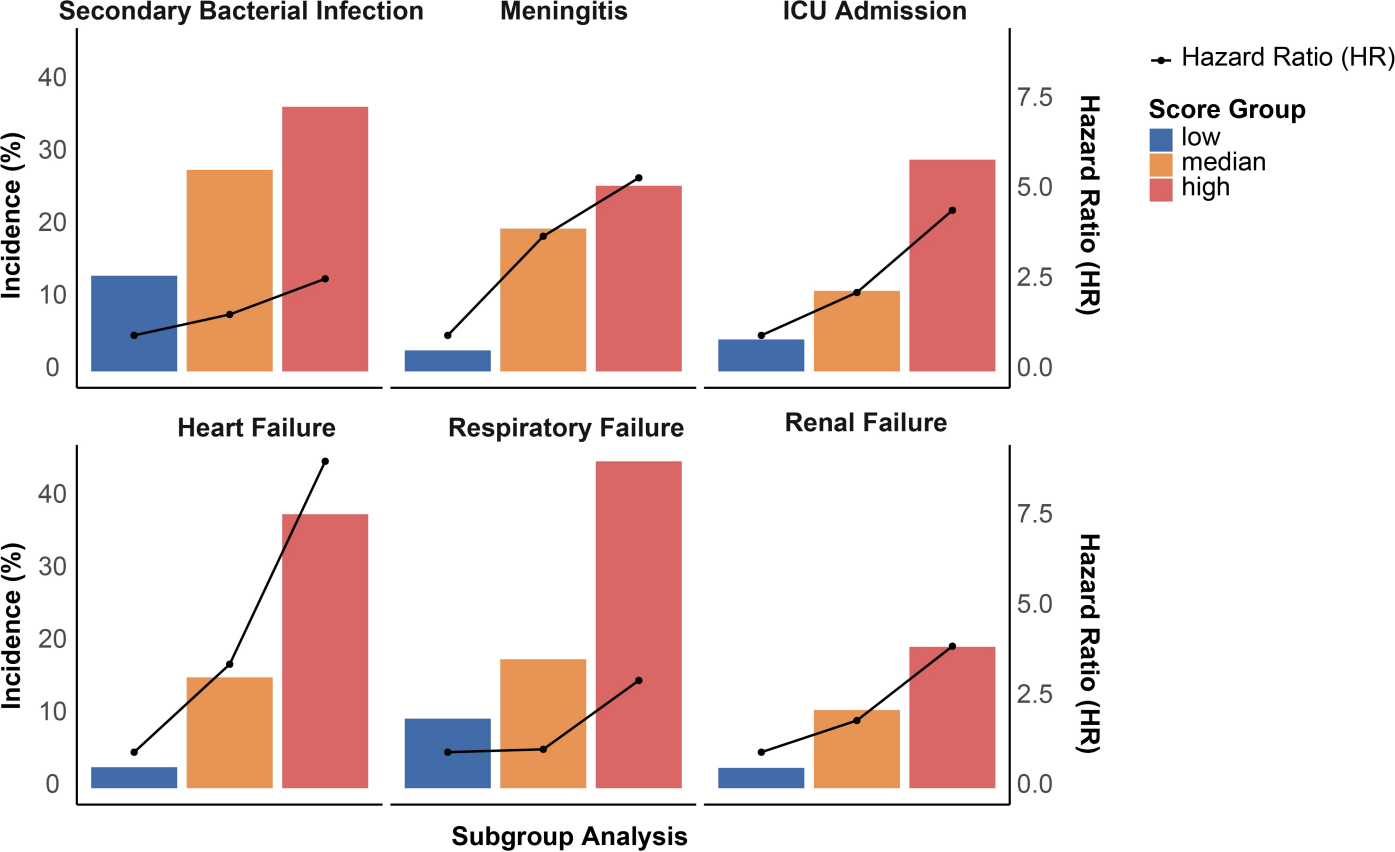
Associations between multi-biomarker score with adverse outcomes among SFTS patients.

A multi-biomarker score was developed, integrating variables of age, AST/ALT ratio, BUN, ALP, and ALB levels (**Figure 2A**). A distinct segregation of scores was observed between participants with survivors and non-survivors, with those in the non-survival group exhibiting significantly higher multi-biomarker scores (P < 0.001; **Figure 2B**). Furthermore, we stratified the multi-biomarker scores into three categories: low (scores 0-1, n = 70), intermediate (scores 2-3, n = 150), and high (scores 4-5, n = 81). Higher score categories were associated with increased mortality risk; specifically, an intermediate multi-biomarker score was correlated with over a 2-fold increased risk of non-survival (HR: 2.34; 95% CI, 0.69-7.90), while a high multi-biomarker score was linked to more than an 8-fold increase in non-survival risk (HR: 8.59; 95% CI, 2.63-28.05; **Figure 2C**). Additionally, Kaplan-Meier survival analysis indicated that mortality varied significantly, ranging from 2.9% in patients with low scores to 40.9% in those with high scores (log-rank P < 0.001; **Figure 2D**). In addition to the association with non-survival outcomes, a high multi-biomarker score was also linked to other adverse clinical events (**Figure 3**), including secondary bacterial infection (HR: 2.57; 95% CI, 1.61-4.21), meningitis (HR: 5.37; 95% CI, 1.24-23.22), ICU admission (HR: 4.47; 95% CI, 2.56-11.14), heart failure (HR: 9.07; 95% CI, 2.15-38.28), respiratory failure (HR: 2.98; 95% CI, 1.31-6.80) and renal failure (HR: 9.07; 95% CI, 2.15-38.28).

### Evaluation of the predictive model

**Figure 4:**
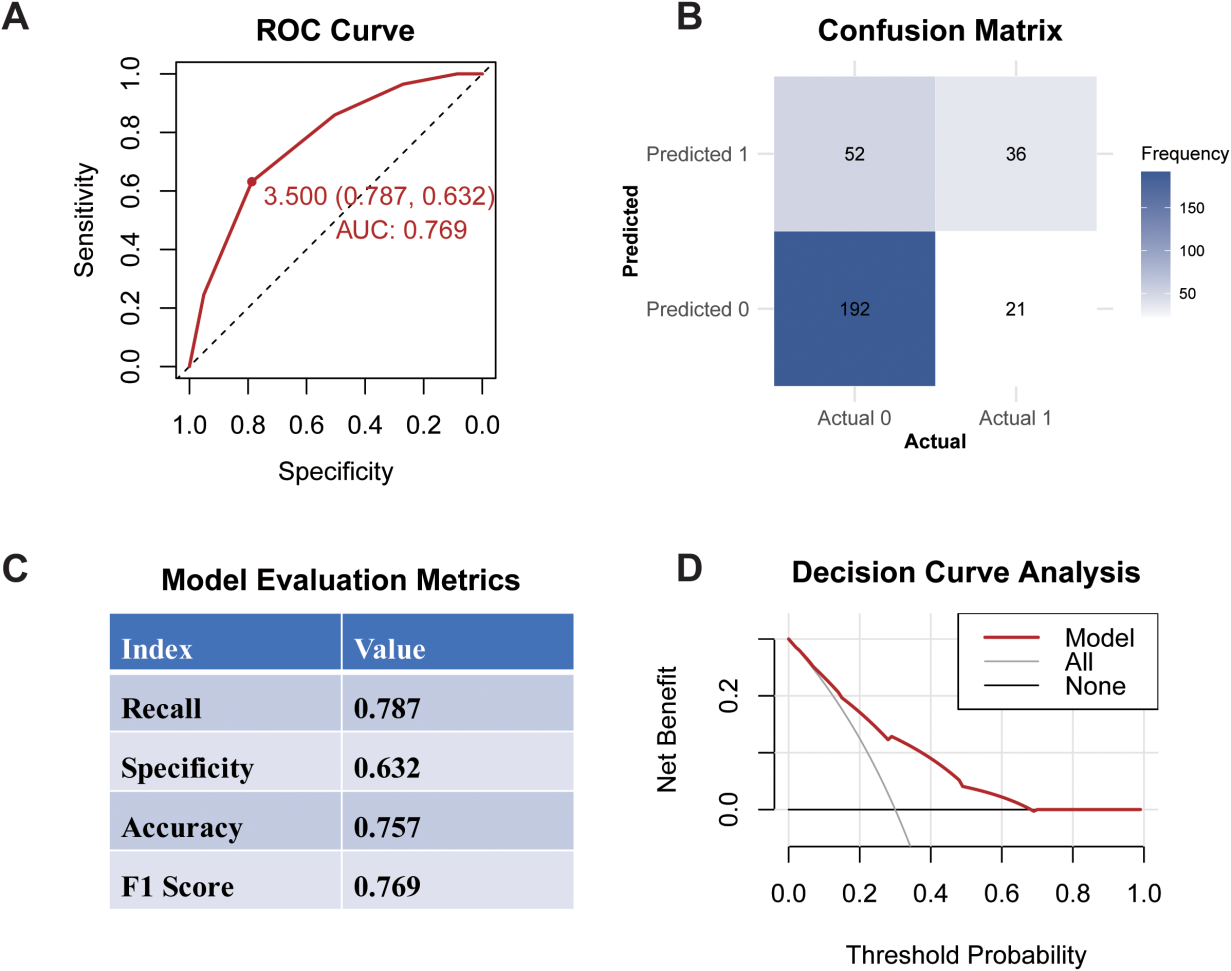
Performance of multi-biomarker score model. **(A)** ROC Curve; **(B)** Confusion Matrix; **(C)** Evaluation Metrics; **(D)** Decision Curve Analysis.

The ROC curve analysis for the developed model yielded an AUC of 0.769 (95% CI: 0.632–0.787), with a recommended cutoff value for the multi-biomarker score established at 3.5, indicating robust discriminative ability (**Figure 4**). The confusion matrix is presented in **Figure 4B**, illustrating the model parameters obtained through ten-fold cross-validation, which included a recall of 0.787, specificity of 0.632, accuracy of 0.757, and an F1 score of 0.769. The Decision Curve Analysis DCA demonstrated that the model’s decision curve is consistently positioned between the ALL curve and None curves across a range of threshold probabilities. This positioning indicates that the multi-biomarker score model offers a significant net benefit, suggesting its considerable clinical utility in decision-making.

### Subgroup and sensitivity analyses

To evaluate the robustness of the multi-biomarker score in predicting survival outcomes in patients with SFTS, we conducted a subgroup analysis. This analysis examined associations stratified by demographic characteristics, medical history, and treatment regimens. Results indicated that a high baseline multi-biomarker score was significantly correlated with an increased risk of non-survival, irrespective of sex, treatment regimen, or the presence of pre-existing hypertension and diabetes (**Table 1**). Furthermore, the multi-biomarker score demonstrated superior predictive performance among females (HR: 2.43; 95% CI 1.57-3.77) and in individuals with comorbid hypertension (HR: 2.71; 95% CI 1.63-4.51) or diabetes (HR: 3.58; 95% CI 1.36-9.40).

**Table 1:** Associations between score and fatal outcomes among different subgroups.

Characteristics	Subgroups	n (*N* = 301)	HR (95% CI)	P value
Sex	Male	147	1.91 (1.39-2.64)	<.001
	Female	154	2.43 (1.57-3.77)	<.001
Living	Local	120	1.72 (1.20-2.48)	0.003
	Foreign	181	2.48 (1.71-3.60)	<.001
Disease history				
Hypertension	No	201	1.84 (1.36-2.49)	<.001
	Yes	100	2.71 (1.63-4.51)	<.001
Diabetes	No	267	1.96 (1.50-2.56)	<.001
	Yes	34	3.58 (1.36-9.40)	0.01
Therapy				
Hormone therapy	No	145	2.59 (1.64-4.11)	<.001
	Yes	156	1.84 (1.33-2.54)	<.001
Antivirus therapy	No	49	1.99 (1.15-3.44)	0.014
	Yes	252	2.10 (1.56-2.82)	<.001
IVIG	No	133	1.95 (1.31-2.91)	<.001
	Yes	168	2.20 (1.57-3.09)	<.001

In the sensitivity analysis, we employed restricted cubic spline regression to identify the optimal cutoff points for each selected biomarker (**Supplementary Figure 2**). Notably, the thresholds for each biomarker and the risk stratification capabilities of the corresponding multi-biomarker algorithm remained largely unchanged. Additionally, we tested the weighted multi-marker score derived from the loading contributions of biomarkers in PCA analysis (**Supplementary Figure 3A**). Similarly, the association between the weighted multi-biomarker score and cumulative mortality risk demonstrated a consistent graded pattern across analyses (log-rank P <.001; **Supplementary Figure 3B**).

## Discussion

In this retrospective analysis, we have developed a multi-biomarker score comprising age, AST/ALT ratio, BUN, ALP, and ALB to estimate the risk of non-survival outcomes in patients with SFTS. Our findings demonstrate that an elevated multi-biomarker score at admission is significantly associated with a higher risk of mortality and severe complications, including heart failure, meningitis, respiratory failure, and renal failure, during the disease progression.

SFTS is a severe infectious disease with a high fatality rate, reportedly up to 30% in China (Li et al., 2021). The virus primarily targets endothelial cells, hepatocytes, and macrophages, leading to widespread endothelial dysfunction, liver injury, and immune dysregulation (Yun-Fa et al., 2025). The infection triggers excessive cytokine release and macrophage activation, contributing to systemic inflammatory response, multi-organ impairment, and coagulation abnormalities (Liu et al., 2021). Given the complex pathophysiology of SFTS, no single biomarker can fully capture the clinical progression trajectory. Therefore, integrating multiple biomarkers that reflect distinct but interconnected biological processes may enhance the accuracy of early risk stratification. Additionally, our multi-biomarker score incorporates five variables identified as high-risk factors for death in SFTS patients: elevated age, AST/ALT ratio, BUN, ALP, and decreased ALB.

Age greater than 60 years was particularly associated with SFTS mortality outcomes, with an adjusted hazard ratio of 3.07 (95% CI 1.46-6.51), aligning with other studies that have identified advanced age as a significant risk factor. For instance, an observational study reported an odds ratio of 3.388 for patients over 65 years old (Liang et al., 2020), and a retrospective study found that each 10-year increase in age raised the odds ratio for death to 1.82 (95% CI 1.62-2.04; p<0.0001) (Li et al., 2018).

The Chinese guidelines for SFTS management highlight the progression of the disease with increasing AST and decreasing ALT levels (General Office of the National Health Commission of the People’s Republic of China and General Office of the National Administration of Traditional Chinese Medicine of the People’s Republic of China). The pattern of elevated AST accompanied by cytopenia resembles the pathophysiological features of macrophage activation syndrome (MAS), in which excessive macrophage activation and cytokine release lead to hepatic injury, hemophagocytosis, and multiorgan dysfunction. Given that macrophages are key target cells for SFTSV replication, similar immune-mediated injury mechanisms may underlie the elevated AST and cytopenia observed in severe SFTS cases (Saijo, 2018). Collectively, studies have also indicated that the AST/ALT ratio, an indicator of hepatocellular injury, serves as an independent risk factor for the prognosis of SFTS (Lianzi et al., 2022). Additionally, elevated BUN has been identified as a significant risk factor for SFTS in clinical studies, while decreased ALB has been associated with death among SFTS patients. Elevated ALP, an indicator of cholestasis, has also been reported as a risk factor for fatal outcomes (Ye et al., 2024). The combination of BUN and ALB, known as the BUN-to-ALB ratio (BAR), has been identified as an independent risk indicator associated with fatal outcomes in SFTS patients (HR: 4.751; 95% CI: 2.208–10.226; P <0.001) (Cao et al., 2024). Our multi-biomarker score introduces ALP into a prognostic model for the first time in the context of SFTS. The inclusion of these biomarkers is supported by their established roles in reflecting hepatic dysfunction and renal impairment, which are critical in the pathophysiology of SFTS. Currently, there is no gold standard predictive model for SFTS patient mortality risk. The indicators used in our study are readily available from routine blood tests and can be used to preliminarily assess patient mortality risk through simple calculations, demonstrating good clinical feasibility.

Nevertheless, our study covered a broad time span from 2012 to 2023. During this period, the COVID-19 pandemic profoundly reshaped global infectious disease patterns and population immunity. The continuous evolution of SARS-CoV-2 variants has been shown to modulate host immune responses and systemic inflammation (Choi et al., 2023), while the rapid advancement and deployment of novel vaccine technologies have influenced baseline immune activation and cytokine profiles in the general population (Jindong et al., 2023). Although our study focused on SFTS, it reflects real-world conditions in which patients’ immune and inflammatory backgrounds may have been shaped by prior viral exposures and vaccination history. Furthermore, emerging evidence indicates that post-acute sequelae of SARS-CoV-2 infection are associated with persistent systemic inflammation, hepatic and renal dysfunction, and immune dysregulation (Ewing et al., 2021; Guo et al., 2021). These immune and metabolic alterations could influence host susceptibility and disease trajectories in other viral infections, including SFTS. In parallel, remarkable advances in diagnostic and pathogen detection technologies over the same period have greatly enhanced laboratory capacity. Improvements in molecular assays, multiplex PCR, and metagenomic sequencing have markedly increased the sensitivity and specificity of pathogen detection. As reported in recent analyses of fever of unknown origin (FUO) in China (Kang and Zheng, 2024), these technological developments have substantially altered pathogen identification patterns and diagnostic efficiency over the past decade. The inclusion of patients across different diagnostic and epidemiological stages enhances the representativeness of our cohort and underscores the robustness and real-world applicability of our model under evolving clinical and laboratory conditions.

This multicenter cohort study offers enhanced applicability compared with existing single-center studies. Moreover, our model provides a comprehensive risk assessment, not only predicting mortality but also evaluating the risk of adverse outcomes such as heart failure, meningitis, and respiratory failure. Clinically, this model is feasible as it incorporates parameters readily obtainable from routine blood tests, offering straightforward calculations and enhanced clinical applicability.

However, our study has several limitations. Firstly, there may be residual or unmeasured confounding factors that could influence the relationship between the multi-biomarker score and the risk of fatal outcomes. For instance, several potentially relevant biomarkers such as ADAMTS13 and prealbumin (PAB) were not included in our dataset, and the associations with disseminated intravascular coagulation (DIC) or thrombotic thrombocytopenic purpura (TTP) were not analyzed due to the limited number of such cases. Future studies with larger and more diverse cohorts are necessary to validate the multi-biomarker score and to explore its potential role in guiding personalized treatment strategies. Secondly, while our study provides population-level insights, the underlying mechanisms and pathways require further investigation.

## Conclusion

SFTS patients with more than three of the following criteria—age ≥60 years, AST/ALT ratio ≥2.23, BUN ≥5.6 mmol/L, ALP ≥68 U/L, or ALB <33.3 g/L—show a significantly elevated mortality risk.

## Data Availability

The raw data supporting the conclusions of this article will be made available by the authors, without undue reservation.
