# Supplementary figures and images for: Multi-biomarker score model for predicting fatal outcomes in severe fever with thrombocytopenia syndrome: a multicenter cohort study
Source: Front Cell Infect Microbiol. 2025 Nov 17;15:1681470. doi: 10.3389/fcimb.2025.1681470 (PMC12665722; doi:10.3389/fcimb.2025.1681470)

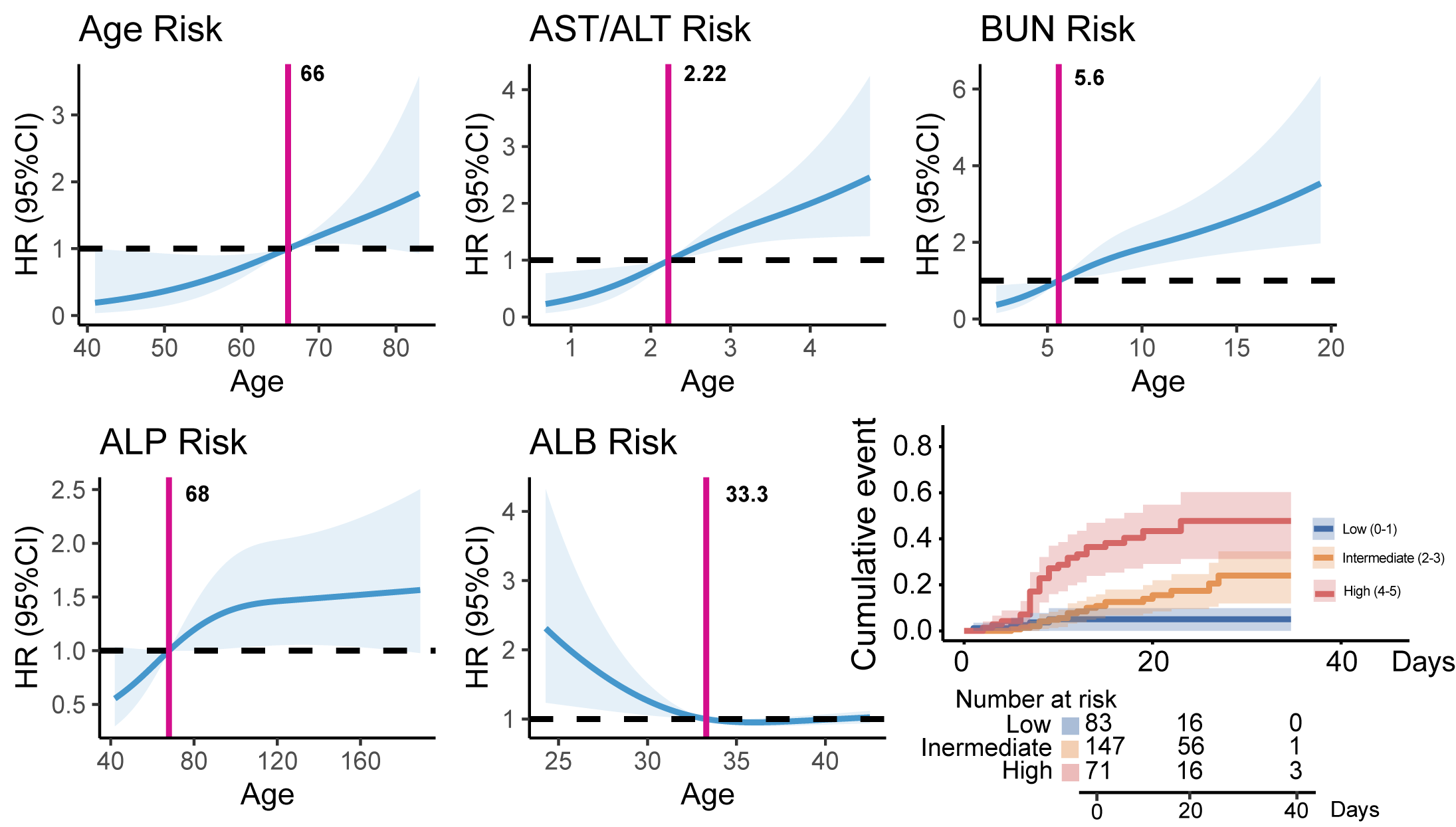

Supplement: Supplementary file 2 [file Image2.tif]

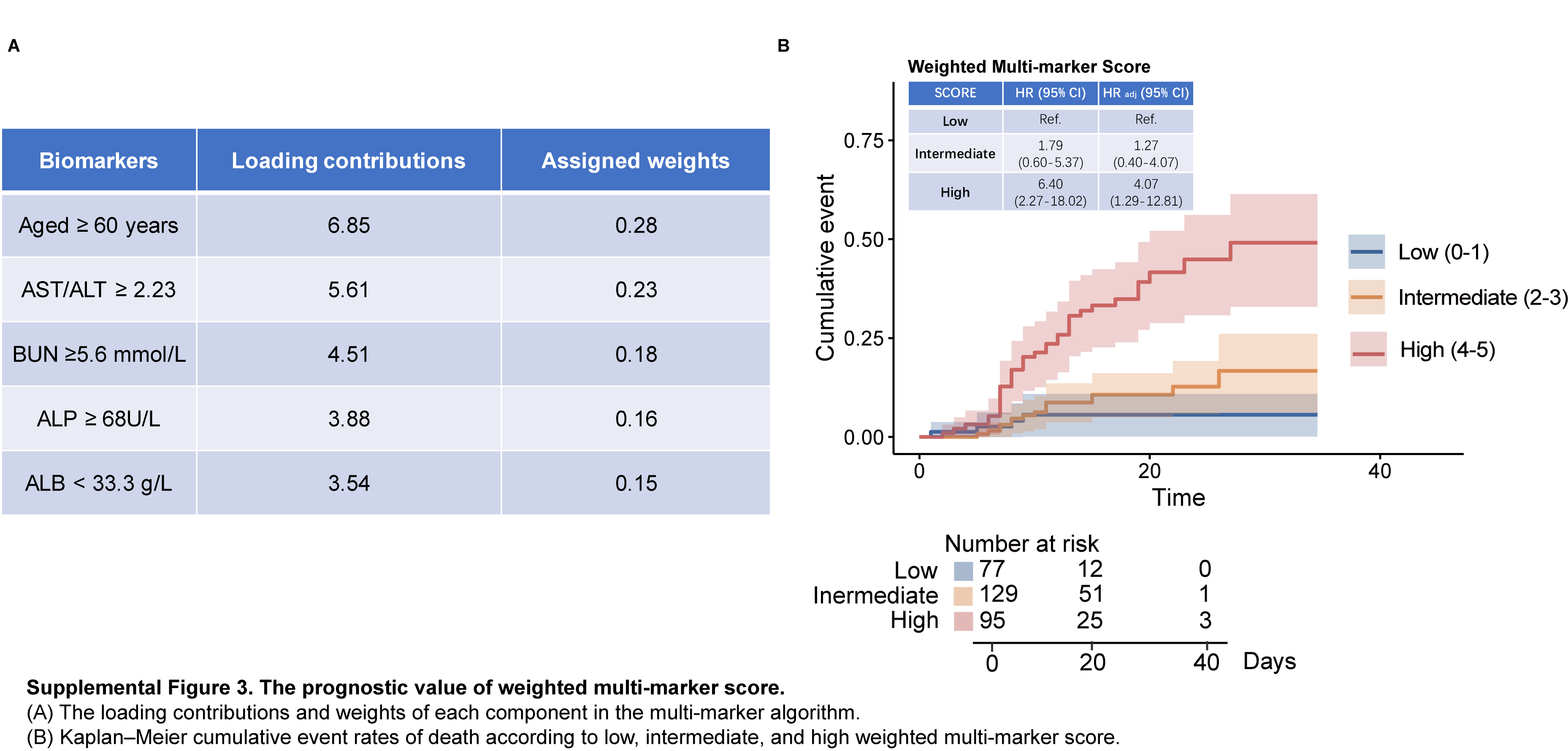

Supplement: Supplementary file 3 [file Image3.tif]
