# Supplementary material for: Multi-biomarker score model for predicting fatal outcomes in severe fever with thrombocytopenia syndrome: a multicenter cohort study
Source: Front Cell Infect Microbiol. 2025 Nov 17;15:1681470. doi: 10.3389/fcimb.2025.1681470 (PMC12665722; doi:10.3389/fcimb.2025.1681470)
Supplement: Supplementary file 4 [file Table1.docx]

| **Supplementary Table 1. Baseline characteristics** | | | | |
| --- | --- | --- | --- | --- |
| **Characteristics** | **Overall (n=301)** | **Cured (n=244)** | **Died (n=57)** | **P value** |
| **Gender** |  |  |  | 0.729 |
| Male | 147 | 121 (82.31) | 26 (17.69) |  |
| Female | 154 | 123 (79.87) | 31 (20.13) |  |
| **Age (Mean, SD)** | 64.9 (11.8) | 63.5 (11.9) | 70.4 (9.5) | <.001 |
| **History of exposure** |  |  |  |  |
| Tick bite or field exposure | 108 | 88 (81.48) | 20 (18.52) | 0.822 |
| Animal contact | 11 | 10 (90.91) | 1 (9.09) | 0.608 |
| SFTSV patients | 13 | 13 (100.00) | 0 (0.00) | 0.142 |
| **Disease history** |  |  |  |  |
| Hypertension | 100 | 77 (77.00) | 23 (23.00) | 0.388 |
| Diabetes | 34 | 28 (82.35) | 6 (17.65) | 0.931 |
| **Therapy** |  |  |  |  |
| Hormone-Yes | 156 | 116 (74.36) | 40 (25.64) | 0.012 |
| Antivirus-Yes | 252 | 204 (80.95) | 48 (19.05) | 0.691 |
